# Human embryonic stem cell science and policy: The case of Iran^☆^

**DOI:** 10.1016/j.socscimed.2013.10.028

**Published:** 2013-12

**Authors:** Mansooreh Saniei

**Affiliations:** School of Social Science and Public Policy, King's College London, United Kingdom

**Keywords:** Human embryonic stem cell science, Policy, Society, Religion, Iran

## Abstract

The paper is based on a large qualitative study of ethics, policy and regulation of human embryonic stem cell (hESC) science in Iran. This case study in five academic research centres used semi-structured interviews to examine in depth the views of stem cell scientists, embryologists and ethics committee members on hESC research policy in this *Shia* Muslim country. Although Iran's policy approach has been considered 'intermediate', what is described here seems to be a 'more flexible' policy on hESC science. This article describes three arguments to explain why Iran has shaped such a policy. These are: (1) a flexibility of the *Shia* tradition has allowed for hESC science; (2) permissive policy related to other fields of biomedicine, such as new assisted reproductive technologies, facilitated approval of hESC research; and (3) a lack of public debate of bioscience in Iran influences how its hESC research policy is perceived. Based on the empirical data, this paper then expands and refines the conceptual bioethical basis for the co-production of science, policy, and society in Iran. The notion of co-production implies that scientists, policy-makers, and sometimes other societal actors cooperate in the exchange, production, and application of knowledge to make science policy.

## Highlights

• Science-policy interface in Iran.
• Iran, Ethics & hESC research.
• Science policy & Islam.

## Introduction

The increasing use of human embryonic stem cell (hESC) lines for research and therapy, as an innovation pathway, has stimulated controversy internationally about the procurement, use and disposal of the embryos. The debate over hESC research is mainly caused by the diversity of ethical and policy issues. Social science, the ethics literature and analysis of policy documents shape discourses, debates, and shifts in this area of bioscience (see, e.g., Franklin, 2005; Isasi & Knoppers, 2006; Parry, 2003; Wainwright, Williams, Michael, Farsides, & Cribb, 2006). Nations across the globe have demonstrated widely divergent levels of tolerance for allowing, funding and regulating hESC science. Taking a 'conservative' approach, some countries allow moral concerns to drive their policy. Other countries take a more 'progressive' approach with centralised regulated systems for hESC science (see Walters, 2004). While several developed countries like the UK, have taken the 'permissive' approach based on long-standing regulation (Isasi & Knoppers, 2006) or specific governance frameworks (Parry, 2006). In developing countries, regulation of this field is a new challenge; responses range from severe restriction to nonspecific or even nonexistent frameworks (Harmon, 2008; Isasi & Knoppers, 2006).

In the Muslim Middle East, only Iran, Turkey and Tunisia have adopted a national policy on hESC science. According to the policy literature, Iran (Walters, 2004) and Turkey (Ozturk Turkmen & Arda, 2008) adopted an 'intermediate' policy that allow researchers to utilise existing hESC lines and use embryos created but not used for *in vitro* fertilisation (IVF). In contrast, Tunisia has banned acquisition of embryos for experimental purposes and allows them to be preserved only for therapeutic purposes, to help infertile couples (Tebourski & Ammar-Elgaaied, 2004).

In 2002 Iran's Supreme Leader, Ayatollah Khamenei, issued a 'stem cell *fatwa*' that declared that experimentation with human embryos was consistent with *Shia* tradition and congratulated the scientists who had produced hESC lines. (A *fatwa* is a religious opinion about whether or not an action is permissible.) Iran's clerics and political leaders have also actively promoted science and technology, in an attempt to enhance the country's international status. With the positive *fatwa* on the use of human embryos for stem cell (SC) research and therapeutic goals, Iran became the first Muslim country to produce, culture and freeze hESCs (see Baharvand et al., 2004). Rapid progress in SC science then led the Iranian government to put in place ethical and scientific supervision of this field of science. Compilation of the Specific National Ethical Guidelines for Biomedical Research (for instance, guidelines for genetic research and gamete and embryo research) has been a major effort in Iran in recent years (Larijani & Zahedi, 2008; Saniei & De Vries, 2008).

The purpose of this article is to briefly present the views of Iranian scientists, embryologists and ethics committee members about Iran's current hESC science policy and to examine some of the reasoning behind their perception. It sheds light on the attitudes of the participants noted above, who are relevant in understanding the status of this field and related policy, in Iran a *Shia* Muslim country.

## Method

Since little has been reported about the views of Iranian scientists and other actors on hESC science, ethics and policy, a case study design was chosen. The case study is an appropriate method when a researcher wants to study a subject in its natural setting and learn about the state of the art (Benbasat, Goldstein, & Mead, 1987). The major research project chosen to be studied integrates data from different sources using multiple methods, including fieldwork, internal archival research, and interviews with 30 senior and junior scientists, embryologists and ethics committee members (15 females and 15 males) associated with hESC science, from five academic SC research centres within Iran (The seniors interviewed can be considered elites in that they all occupy positions in power networks). To validate claims about the sites, an online search was conducted for other sources to see if the findings from interview data could be corroborated and to identify any contradictory evidence (e.g., about the ethical guidelines on hESC research).

Following approvals by ethics committee at King's College London and the Royan Institute in Iran, 'purposive' and 'snowball' sampling (Silverman, 2010) was done for maximum variation of ideas and perceptions, with the aim of recruiting enough numbers to reach thematic saturation. Interviewees were recruited using both formal and informal approaches and were given oral explanations and information sheets describing the research. Between March and May 2010 the interviews were conducted in Persian as guided conversations (Lofland & Lofland, 1984) lasting 55-170 min (average 95); an in-depth, semi-structured format allowed interviewees to use their own words and shape the discussion in ways related to their experience. None of the respondents objected to their interviews being quoted anonymously. Broad sets of questions covered interviewees' understanding of hESC science and policy and their general awareness of the contextual factors shaping the field.

All interviews were fully transcribed and then translated into English by the author, who is fluent in both languages, and a thematic approach was used to analyse the transcripts. The analysis and the themes that emerged were discussed with the author's supervisors and colleagues. This paper quotes from interviewees involved in science policy-making (12 interviews from 3 sites). It is important to note that though the quotes are typical of those interviewed for this study; they may not be generalisable to the wider scientific and academic community in Iran. To preserve anonymity, study numbers were assigned and reference to occupations is in general terms rather than by specific job titles. For instance, the category of 'scientist' could be used for those involved in producing the lines and for those who differentiate them into different kinds of cells.

## Results

### Themes

This analysis is organised into two major themes, each with several subthemes to help explain the overall findings of Iran's current policy on hESC science. Each theme corresponds to embedded, inter-related ethical, social, legal and religious debates over Iran's hESC science policy.

#### (Un)certain hESC science policy

Several different perceptions of hESC policy emerged in the interviews, in which some participants also expressed views that spanned more than one subtheme. Their comments mainly refer to Iran's current policy for hESC research, such as encouraging, more flexible, liberal and/or open-minded.

##### Encouraging

The first category is closely aligned with the official *fatwa* from Iran's Supreme Leader:

*They [the Iranian government] somehow encourage [scientists] to do this kind of [research]. This field, it seems, can respond to many human problems, related to [medical] disorders, war-damaged people, and many scientific questions. (Scientist/7/Male)*

Another interviewee added further details to this quote in explaining that:

*[Ethics policy] doesn't restrict our research activity. Our country fortunately emphasises the importance of improving research, discovering human creation, and helping patients. (Scientist/1/Male)*

Several interviewees said that it had been encouraging when Ayatollah Khamenei publicly supported the field in 2002. For the Supreme Leader, the main reason for doing hESC research was its 'global benefit' for human beings-he encouraged scientists to advance the technology to save lives, considering it a religious duty to carry out research in order to develop new medicines and technologies that can benefit humanity. Iran's goal should also be to become the 'leader of science' in the Middle East in the next 20 years (Khamenei, 2007). The scientific progress of Iranian science has been demonstrated by Rudolf Jaenisch's paper published in *Nature* (Jaenisch, 2007) as well as a stream of articles in *Science* and *Nature* about Iran's progress in science. Dr. Baharvand, the head of the Department of Stem Cell Research at the Royan Institute, stated: *"[The] vision is to efficiently put stem cell research findings into operation in disease treatment to increase the level of health."* (Morrison & Khademhosseini, 2006: 8). The task of scientists, then, is to promote scientific progress and keep Iran on the leading edge of discovery.

##### More flexible

With its 'permissive', 'flexible' and 'restrictive' policies on hESC research, Iran has adopted a 'flexible' policy, among the categories described by Hoffman (2008), though some other references named it as an 'intermediate' approach (see Isasi & Knoppers, 2006; Walters, 2004). Adhering to this policy, Iranian scientists would work only on 'spare' IVF embryos and they were not allowed to generate embryos for research purposes. However, a few interviewees considered the policy to be 'open-minded'. For instance, one ethics committee member noted that:

*We should go towards treating human by this method [hESC research and therapy] but we have to consider the ethical debates. This is our duty. There is no prohibition [on hESC research] in Iran and our policy is open-minded. Our research institutes are very active in hESC science to find the cure for debilitating diseases. (Theologian/Ethics Committee Member/30/Male)*

Several interviewees also drew attention to the use of the somatic cell nuclear transfer (SCNT) technique to provide SCs for therapeutic purposes. One of the embryologists argued that:

*We use SCNT in animal research and plan to carry out this technique for obtaining human stem cells which are genetically matched to the donor organism. (Embryologists/Ethics Committee Member/26/Male)*

Another one noted:

*I think that all kinds of stem cell research, including therapeutic cloning, should be encouraged. In my view therapeutic cloning is a different matter [from reproductive cloning]. [...] It would be indefensible to stop this research and deny people [patients] the chance of new treatments which could save their lives. (Physician/Ethics Committee Member/2/Male)*

These quotes reflect the idea that the total prohibition of human cloning was unacceptable and would block valuable research and medical advances in treating debilitating conditions, such as cancer, Alzheimer's and Parkinson's diseases, spinal cord injuries and so on.

The Supreme Leader often cites the *Qur'an*'s emphasis on preventing human illness and suffering as evidence that SC research and Islam are compatible; the *fatwas* permitting biomedical research in Iran are the product of such science-friendly interpretations. Iran's stem cell *fatwa* stresses the admissibility of destroying spare IVF embryos in order to collect SCs for research. However, there are limits: the Supreme Leader has warned Iranian scientists *"to be careful that producing identical parts of human beings does not lead to producing a human being."* (Iran News, 2013) Human reproductive cloning is not accepted by many Muslim scholars mainly because it could be considered misuse of the woman who supplied the eggs. There is also a special concern that any human born from such an experiment would be more likely to suffer from impaired health and development. Moreover, some Muslim scholars hold that the loss of kinship and lineage as a result of the unnaturalness of reproductive cloning, as well as potential social harms (Larijani & Zahedi, 2004).

Authorities in Iran tend to accept therapeutic cloning to produce SCs, according to Larijani and Zahedi (2008:631): *"The Islamic view about when life begins has been used by Iranian jurists for issuing fatwas on allowing stem cell research and cloning for therapeutic purposes."* This policy is in favour of the potential benefits of hESC and therapeutic/research (but not reproductive) cloning. Iran's *Ethical Guidelines on Human Embryonic Stem Cell Research and Therapy*, drafted by Nejad-sarvari, Emami-razavi, Larijani, and Zahedi (2011) implies this policy on therapeutic cloning. However, it is not permissible to transfer human somatic cell nuclear into animal eggs.

Although the policy is seen as 'open-minded', some interviewees emphasised the limits to flexibility. In this quote an interviewees argued that:

*It might seem that Iran is open-minded. We might not have so much limitation [in working on human embryos], but we're not allowed to create embryos for research, or animal-human hybrid for therapeutic purposes. (Embryologist/20/Female)*

One scientist also added, "*I don't think that Iran is flexible. I think, they work on this field of research, because they have a religious justification and perhaps scientific interest."* (Scientist/8/Female) Another scientist agreed, saying, *"The country considers [hESC research] as opportunity. Sometimes, I don't think that they're too permissive, but they're doing this research because it's religiously justified.*" (Scientist/7/Male) One interviewee who considered Iran's policy to be 'restrictive' explained, *"Islam has a careful definition of full human beings, and based on this definition, I can say that Iran is very strict about the use of human embryos for research purposes."* (Scientist/27/Male)

#### Perceptions of Iran's hESC science policy

##### Shia tradition and bioscience

The ontological status of pre-implantation embryos is the most sensitive point in the long-running dispute over hESC research. One concrete illustration of the influence of ideology on bioscience and policy-making is the use of embryos for SC research. As many interviewees noted, one of the main reasons for this progressive approach is the role of *Shia* tradition in supporting hESC science. One scientist argued that Iran's open-minded policy comes out of the *Shia* tradition which is compatible with the development of science and said:

*It [open-minded policy] is related to our beliefs. Shia [tradition] says, "No problem, you can work on a human embryo," which is before ensoulment [the creation of a soul within a human being]. The embryo has potential to become a human being but it's not yet [a human being], [it] doesn't have soul yet, so it's allowed [to be used]. (Scientist/3/Male)*

For interviewee 3, Iran's policy emerged from its people's religious beliefs, which are based on in-depth examination of what constitutes the moral status of human embryos, and whether the practice of hESC research with all its ramifications would violate human dignity. However, many Muslim scholars, both *Sunni* and *Shia,* have approved creation of hESC lines for research and therapeutic use, even if that requires destruction of surplus IVF embryos, because very few of the embryos will have the chance to develop into mature human beings (Sachedina, 2009). The more plausible view, for Muslim jurists, is that because human entities at the beginning of life do not have a moral status, they can be used for research purposes.

Several interviewees emphasised the importance of this religious justification and its role in hESC science and policy in Iran. One embryologist 25, who was a member of an ethics committee said, "In Islam and particularly [in the] Shia [tradition], we can see this open-minded approach to many new matters [e.g., therapeutic abortion]. Shia doesn't restrict us to the limited action." Scientist 17 added, *"Even, in religious debates, we are different from other [Muslim] countries. We are the only country with the Shia system [because of the large Shia community], and take everything easier than Sunni."*

Despite its reputation in the West for intolerant Islamic fundamentalism, Iran has relatively permissive legislation in several areas of biomedicine, such as abortion, assisted reproductive technologies (ARTs) and hESC research. Larijani and Zahedi (2006) said that *Shia* Islam teaches that ensoulment takes place at 120 days after conception (see also Saniei, 2012a, for more information about Islam and embryonic development). This makes it possible for Iranian physicians to do therapeutic abortions and for scientists to perform hESC research and therapeutic cloning.

In 2005, the Iranian parliament passed a 'therapeutic abortion act', which allows abortion after a diagnosis by three experts and confirmation by the 'legal medicine organisation'. Abortion is now legal during the first four months of pregnancy in case of mentally and physically handicapped foetuses (Bazmi, Behnoush, Kiani, & Bazmi, 2008). According to Larijani and Zahedi (2006) the incidence of birth defects seems to be rising in Iran. There is a preference for consanguineous marriage, which results in a higher level of defects and, as in the West, the age of marriage for educated women is rising in the country.

As noted earlier, Iran's approach to biomedical advances and science policy has been remarkably progressive in most area, but also controversial in many. Why does Iran take an approach to these issues that is different from approaches in other parts of the Muslim world? One interviewee argued that the main reason can be *Shia* jurisprudence, because it utilises *ijtehad* (in Islamic law, the independent or original interpretation of problems not precisely covered by the *Qur'an* and the Prophet's sayings) by adopting reasoned argumentation in finding the laws of Islam. He then stated:

*Shia fiqh (jurisprudence) is more dynamic than Sunni fiqh, as we use aql (intellect or reason) and Sunni rely more on strict readings of Islamic texts. It's our duty to use aql and be flexible. Islam always emphasise science. It's very important for Muslims. Since the time of the Prophet, many things have been discovered, and we should adapt to them. It's our duty to advance knowledge. (Theologian/Ethics Committee Member/30/Male)*

As a *Shia* Islamic country, Iran is influenced by a culturally-based religious faith. While scientific and technological advances continue to provide new challenges, *Shia* jurists have used several principles of Islamic jurisprudence to find valid solutions without breaking Islamic law (Saniei, 2012a). For instance, legal principles such as *maslahat* (public good) and *istihsan* (to deem something preferable) promote what is beneficial; and z*arurat* (necessity), which overrules prohibition, might provide religious-legal justification and legitimisation (Sachedina, 2009). Senior *Shia* scholars applied the principles of *ijtehad* and *ijma'* (scholarly consensus) and concluded that hESC research is permitted in certain circumstances, e.g., the use of pre-implantation and pre-ensoulment embryos (Saniei, 2012a).

Another participant added:

*For us [as Shia], the difference is we can be more flexible in using new science, as we can adapt the religious texts to modern society through our own interpretations and reason. Because Islam, particularly Shia Islam, emphasises science and new thinking, there is no challenge between religion and science. When new things come, Islam is able to accommodate. (Embryologist/Ethics Committee Member/26/Male)*

These two quotes resonate with a view widely held in Iran about the perceived flexibility of *Shia* tradition compared to *Sunni.* That is not to say that other Muslims do not also use various methods of logic in Islamic *fiqh*, but Iranians definitely perceive their own system as more flexible and dynamic when it comes to incorporating scientific advances into daily life. This flexibility of religious institutions vis-a-vis hESC science thus originates from the perception that *Shia* allows adjustments to accommodate changes within Islamic beliefs, while the *Sunni* doctrine is more strict. This automatically allows the Iranian scientific community, where *Shia* prevails, to have a comparative advantage over those in neighbouring countries because they are permitted to work with hESCs (see Saniei, 2012b for comparative policies among selected Muslim nations).

##### Analogy to the new assisted reproductive technologies

The diversity of opinions on hESC science policy sometimes connects to the complex views about what Iran's policy is in other fields of bioscience and technology. Some interviewees commented about the goals and practices of other areas of biomedicine, for instance, the new ARTs, such as third-party gamete and embryo donation. One interviewee, in discussing bioscience policy said that:

*Of 57 Muslim nations, Iran is the only country which has surrogacy and third-party egg, sperm and embryo donation. Iran thinks about sex selection. We also have stem cell [therapy], [animal] cloning, and PGD [pre-implantation genetic diagnosis]. The reason behind the ARTs policy is the knowledge of our Ulama [also called Maraji]*^1^*about the Islamic debates. According to them, "Everything is permitted, unless there is a reason against that." (Physician/Ethics Committee Member/2/Male)*

Another interviewee also drew attention to this subject and argued that:

*Our Ulama carefully assess debates surrounding hESC research. If you look at similar research, such as third-party embryo donation, you can find that Iran precisely works based on religious discourse and deliberation about ethics and bioscience. (Scientist/1/Male)*

The endorsement and support of the religious authorities has thus made the use of ARTs possible among the Muslims in the Middle East. Marcia Inhorn (2006a) notes that the global spread of these technologies is nowhere more evident than in the 22 nations of the Muslim Middle East. In most Muslim countries, however, application of these technologies is limited to IVF treatment only for married couples; and third-party donation is not permitted (Inhorn, 2006a). *Shia* Iran, as a theocratic state, has adopted all forms of the new ARTs and has legitimised them, including third-party gamete and embryo donation, surrogacy and sex selection (Tremayne, 2009). With regard to Iran's policy on ARTs, Inhorn (2006b) says, *"Iran is definitely in the lead among Muslim countries in the Middle East in the application of these technologies."* The reasons for and the process of legitimising the new ARTs in the Muslim Middle East have been documented extensively elsewhere (Inhorn, 2006a; Tremayne, 2009). However, there is no religious *fatwa*, governmental act or clarification in Iran's biomedical guidelines about sex selection.

Interviewees brought up this example to draw attention to Iran's open-minded policy on other fields of medical technology, such as embryo donation for infertility treatment. By making an analogy between new ARTs and hESC research, they attempt to demonstrate how the state policy has been applied to a variety of subjects. In doing so, in response to this progressive approach, the scientific community has shifted the ethical, social and legal focus from hESC research to ARTs. It seems that the similarity between hESC research and other bioscience fields like third-party embryo donation helps interviewees to see Iran's policy as 'more flexible' and 'open-minded' and unlike the policies of most other Muslim countries.

##### Lack of public debate

In contrast to many countries, public opinion in Islamic countries like Iran, does not much influence on these types of issues. Aramesh and Dabbagh (2007) explain that the rules, regulations and practice in Iran are mainly based on *fatwas*, which do not grow out of public and secular debate. Several interviewees pointed out that the lack of public debate could be one reason why Iran's policy seems 'open-minded'. For example, in the following interview one embryologist explained that:

*It seems that Iran is so liberal [in terms of science]. [...] The public don't criticise us, and this might be the reason why they [other countries] think that we're liberal. It means that we don't have any sociocultural barriers. If we don't inform them [the public] [about our scientific activities], no one [asks any question or] stops us. Even journalists don't criticise us. (Embryologist/20/Female)*

Similarly to this view another embryologist presented the following argument:

*I don't agree that [Iranian] scientists are free. In Iran, when scientific work progresses a little bit, for instance, based on individual interest, the public might criticise them; [...] then [regulators] look for a ruling about that (Embryologist/29/Female).*

Taken together, these quotes may reflect the opinion that the absence of public debate leads scientists, policy-makers and advisory bodies to make defensive assumptions about what the public might find acceptable.

Some Islamic states oversee all aspects of life and sometimes prevent the public from expressing their opinions, so the general public has little influence on decisions made by politicians. Iran has a centralised *Shia* authority, represented by the Grand Ayatollah, who has the most religious and legislative power in the country. The importance of this fact is that assessment and practice of any subject are based upon religion, which more readily provides clear and direct information about the country's approaches to embryo research, at least from a *Shia* perspective. Accordingly, once a procedure has been allowed, scientists sometimes operate with no accountability. This religious constitution, however, facilitates and speeds up the policy- and decision-making process. It can even allow for research when there is no legal regulation on a certain subject (Saniei, 2012a). It seems that hESC research has the possibility of a promising national and internationally future for regenerative medicine in Iran (Khamenei, 2007). Moreover, in Iran the trend seems to point to growing optimism among patients towards SC research (see Javadi et al., 2005); and thus the laws are open to research on cell lines developed from embryonic cells.

## Discussion and conclusion

The emergence of major advances in biomedical technologies, such as gamete and embryo donation, genetic testing, and SC research and therapy, have led to redefinition of what constitutes life. In the West, the emotional and ethical disputes surrounding these technologies have been substantial. Throughout the Muslim world, the response has been equally fraught with moral and religious concerns, and with a similar lack of consensus. *Fatwas* on these modern debates vary among the different Islamic schools of thought; these Islamic rulings actually reflect local customs, cultures and moral sentiments. Marcia Inhorn's (2010) study comparing the *Sunni* and *Shia* approaches to gamete donation and infertility treatment in Egypt and Lebanon pointed to how different Islamic interpretations of what is permitted influence government reproductive policies and individual practices.

Individual countries have adopted different policies on the use of embryos for SC research based on their sociocultural, political, and even economic backgrounds. Isasi and Knoppers (2006: 9) noted that "the historical, cultural and sociological context, the institutional framework, and the mobilisation of stakeholders are factors that help explain why countries that seemingly share similar socio-religious beliefs [and perhaps scientific interests] have adopted diametrically opposite public policies." Countries with similar religious backgrounds and, perhaps, scientific interests may therefore approach new knowledge in different ways as a result of the mutual influence of cultural factors and science.

This paper adopts an interpretive approach to the science, society and policy, which are understood as historically and culturally situated (Haraway, 1991) and co-produced (Jasanoff, 2004) along with the social contexts involved in their making. For Iran, introduction of an Islamic system seems to have forced religious scholars into an unprecedented role of responsibility and involvement in social planning and public health. Having been faced with health crises on a large scale may partially explain why religious scholars have invoked *maslahat* and *istihsan* in their rulings on medical and health affairs rather than considering these questions in an isolated or theoretical sense, as was done in the past. The financial burden of debilitating diseases is also central to decisions about hESC research in Iran. This may have given *Shia* scholars impetus to reconsider the public health ramifications of degenerative and incurable disorders or the financial hardships that serious, long-term illnesses have on individuals, families and society. The eight-year Iran-Iraq war left the country with a large community of people disabled by spinal cord injuries, among others, a fact that provided intensive motivation for Iran to start many cell-therapy research projects (see e.g. Javadi et al., 2005). In developing countries (e.g. Iran), domestic cell therapy and regenerative medicine is also a low-cost solution for the growing number of patients with chronic diseases, including diabetes, heart diseases, hepatitis, and such blood diseases as thalassaemia, which are relatively prevalent (Greenwood et al., 2006).

Along with high demand, a combination of surging science, encouraging policies, and favourable ethical norms that make spare embryos accessible for scientific research (Inhorn & Birenbaum-Carmeli, 2008) are all contributing to push innovation forward. Surely, that move could not have been accomplished without interaction between scientists and non-scientists-policy-makers and other actors who have an interest in hESC and the policies that affect it. Furthermore, technological innovation and scientific achievement holds hope of a 'golden age' of intellectual and material benefits for the people of Iran, as the Supreme Leader asserted in justifying the position of the country in the Muslim world.

This paper uses the case of hESC science in Iran as a vehicle to explore the politics of innovation that shaped the co-production of science, policy and society. The use of human embryos in hESC research has raised fundamental sociocultural, moral and religious issues about the relationship between human life and scientific progress: issues that may collide with science policy and regulation. It is important that this collision be resolved in terms of how science policy is constructed and understood, the authorities on which it draws for legitimacy in its consideration of possible science-culture conflict, and the sustainability of the policy outcome given the range of actors involved.

Different perceptions of hESC science policy are constructed in different ways. Based on the dominant narratives, this paper argues that Iran's policy on hESC research is 'more flexible', 'open-minded' and 'encouraging' rather than simply an 'intermediate' approach; and the science is closely intertwined with ethical, social, religious and legal considerations. It contributes to the development of a more socially embedded account of ethical deliberation about hESC science policies and their effects and also to the social and policy contexts of hESC research ethics. As this study continues, to date, no systematic variation has been found in interviewee opinions on ethical, social and legal issues in relation to their main scientific interests and religious convictions.

## Footnotes

1 *Ulama* (singular: Alim, 'scholar') or *Maraji* (singular: *Marja*) are the supreme legal authorities and the source of emulation, for Twelver *Shia* Muslims (followers of the twelve Imams, the Prophet's offspring) around the world. The concept of *Marja-i Taqlid* (lit. source of emulation) is central to *Usuli* (principles) *Shia* Islam (see http://www.globalsecurity.org/military/intro/islam-ithna-ashari2.htm). The *Marja-i Taqlid* provides religious interpretations of matters of law and rituals. Ideally, the most just and knowledgeable specialist in the field of the Islamic law should become recognized throughout the Muslim world as the *Marja-i Taqlid*. In practice, however, this rarely happens, so there are several *Marja-i Taqlid's* which an individual is free to choose and emulate. Those clerics who reach the apex in the theological hierarchy in the centres of *Shia* learning become *Ulama* or *Marja-i Taqlids.*
